# Salivary exosomal microRNAs as biomarkers for head and neck cancer detection—a literature review

**DOI:** 10.1186/s40902-021-00303-9

**Published:** 2021-06-30

**Authors:** Cosmin Ioan Faur, Horatiu Rotaru, Ciprian Osan, Ancuta Jurj, Rares Calin Roman, Madalina Moldovan, Magdalena Chirila, Mihaela Hedesiu

**Affiliations:** 1 Department of Oral Radiology, “Iuliu Hatieganu” University of Medicine and Pharmacy, 400033 Cluj-Napoca, Romania; 2 Department of Oral and Cranio-Maxillofacial Surgery, “Iuliu Hatieganu” University of Medicine and Pharmacy, 400033 Cluj-Napoca, Romania; 3 Faculty of Dental Medicine, “Iuliu Hatieganu” University of Medicine and Pharmacy, 400033 Cluj-Napoca, Romania; 4 Research Center for Functional Genomics, Biomedicine and Translational Medicine, “Iuliu Hatieganu” University of Medicine and Pharmacy, 400337 Cluj-Napoca, Romania; 5 Department of Otorhinolaryngology, “Iuliu Hatieganu” University of Medicine and Pharmacy, Cluj-Napoca, 400000 Romania

**Keywords:** HNSCC, Exosome, Extracellular microRNA, Biomarker, Saliva

## Abstract

**Background:**

MicroRNAs (miRs) are small, non-coding mRNA molecules which regulate cellular processes in tumorigenesis. miRs were discovered in extracellular environment and biological fluids, carrying marks of head and neck squamous cell carcinoma (HNSCC). They were also identified in abundance in salivary exosomes, in which they are protected by exosome lipid barrier against enzymatic injuries and therefore, the accuracy of exosomal miR-based cancer detection increase. This systematic review aimed to reveal and inventorize the most reliable exosomal miRNAs in saliva samples which can be used as novel biomarkers for early detection of HNSCC.

**Materials and methods:**

A systematic literature search, according to PRISMA guideline, was performed on Pubmed and Google Academic libraries, based on specific keywords. Original articles published between 2010 and 2021 were selected. The quality of each paper was assessed using the Quality Evaluation Scoring Tool.

**Results:**

At the end of selection process, five studies met the inclusion criteria. These studies analyzed twelve salivary exosomal miRs, presenting different methods of exosome and miR identification for HNSCC detection. A comprehensive explanation of the miR pathways of action was drawn and illustrated in this review.

**Conclusion:**

Exosomal miRs are promising biomarkers for oral cavity and oropharyngeal cancer detection. miR-10b-5p, miR-486-5p, miR-24-3p and miR-200a stand as the most useful ones in saliva sample examination.

## Background

Affecting a large variety of anatomic subsites such as oral cavity, pharynx, larynx, salivary glands, nasal fossa and paranasal sinuses, early and non-invasive head and neck cancer detection is a challenging topic [1]. The most frequent outset origin is from squamous cells of the epithelium, but the anarchic proliferation of mesenchymal or neural cells could also be involved. The annual incidence of head and neck squamous cell carcinoma (HNSCC) is more than 900,000 new cases worldwide, and it is greatly correlated with tobacco smoking and chewing, alcohol consumption and HPV infection [2, 3]. Despite the considerable diversification of therapeutic methods, long-term survival rate remains under 50% due to the late diagnosis, frequent onset of multiple primary tumors and tumor relapse [4]. At initial presentation, more than 40% of patients are found with regional nodal involvement (HNSCC stage IVA or B), and 10% are diagnosed with distant metastases (HNSCC stage IVC), correlated with a poor prognostic [5]. Therefore, the interest in discovering new methods for early cancer detection is very high, promoting immediate therapy to increase patient overall survival rate and quality of life.

The exosomes are 50–150-nm extracellular vesicles (EV) involved in the intercellular communication. Exosome biogenesis is a complex process which involves 3 main parts: endosome formation by the inward budding of the plasmatic membrane, the generation of multivesicular bodies (MVB) containing intraluminal vesicles and the fusion between MVBs and cellular membrane, resulting in the release of the exosomes in the extracellular environment [6]. The structure of this EV is related to the original cell, being composed of a lipidic double layer that surrounds the internal components: proteins, lipids, DNA, RNA and miRs. In addition, exosomes possess specific surface proteins that can be used for differentiation between these nanoparticles and other microvesicles or apoptotic bodies [7]. These membrane proteins, such as tetraspanin CD81 or CD9 allow sorting, selective recruitment and profiling of the cancer cell-derived exosomes [8].

MicroRNAs (miRs) are ~ 22-nucleotide small non-coding RNAs that interact with the RNA-induced silencing complex (RISC), binding to the 3’ untranslated region (UTR) of mRNA to induce either mRNA degradation or mRNA translation inhibition into specific proteins [9]. Regulating the target gene expression, miRs influence vital processes including cell cycle, apoptosis, proliferation and differentiation. Moreover, any dysregulation of miR expression may contribute to the outset, development and invasion of various types of cancer, including HNSCC [10]. Besides the cytoplasmatic miRs, a considerable number of scientific research highlighted the ability of this molecule to survive in extracellular environment. According to recent studies, there are 4 primordial mechanisms of miR secretion into extracellular space: through exosomes, shedding vesicles, apoptotic bodies and in association with proteins or high-density lipoproteins [11]. Being packed into exosomes, miRs are protected from enzymatic degradation against endogenous RNase. As a result, they are more stable in the extracellular environment compared with cell-free miRs. Exosomal miRs can be identified in body fluids, such as blood and saliva, making them optimal candidate biomarkers for malignant tumors. Several miRs, such as miR-3714, miR-650 and miR-575, were identified significantly altered in the saliva of nasopharyngeal carcinoma patients comparing with healthy controls, which emphasize the opportunity of using salivary miRs as a method of diagnosis [12].

Despite of the abundance of cancer biomarkers identified recently in saliva, none of them was certified as an indisputable fingerprint of malignancy. Moreover, exosome isolation and characterization, as well as exosomal miR expression quantification, have various methods of investigation which could induce heterogeneous results.

The purpose of this review is to spread light over this biomarker-based diagnostic method and to identify the most reliable exosomal microRNAs in saliva as an early, effective, and non-invasive diagnosis tool for HNSCC.

## Materials and method

### Publication search

A search on Pubmed and Google Academic databases was performed according to PRISMA methodology 2015 Checklist algorithm for systematic reviews and meta-analysis [13]. The strategy for literature search was represented by “((saliva and exosome) or (saliva and microRNA) or (exosome and microRNA)) and (head and neck cancer)” on Pubmed.gov and “salivary exosomal microRNA” on Google Academic.

### Inclusion and exclusion criteria

A year publication filter was applied for 2010–2021 interval of time. The studies selected from both databases were original articles only. An original article was defined as a research performed on clinical subjects or a laboratory research which presents original data. Reviews and meta-analysis were excluded. Duplicated papers were also removed. The abstract and full text were further analyzed. The original articles which did not focus on the topic of head and neck cancer detection or premalignant lesion detection using salivary exosomal miR as biomarkers were excluded. In the following stage, the eligibility criteria of the studies were applied, as listed below:

1. Availability of the text in full format;
2. Head and neck cancer or premalignant lesion detection or management;
3. Saliva samples from patients and control as material for research;
4. Salivary exosomal microRNA;
5. Quantification of the parameters of test in terms of sensitivity, specificity, AUC or ROC;
6. Results presented as quantitative data;
7. Article written in English;
8. Respecting the structure of IMRAD (introduction, material and method, results, discussions).

Each article that did not respect all of the criteria was excluded from the full-text analysis.

### Qualitative assessment

The QUEST quality assessment tool was used to evaluate all studies included in this systematic review. This tool comprises of 6 quality parameters: Authorship, Attribution, Conflict of interest, Currency, Complementarity and Tone. Each article was scored from 0 to 28 according to the above-mentioned parameters, a higher value indicating a reliable methodology. To diminish the risk of bias, two distinct reviewers analyzed the selected articles using the Quality Assessment of Diagnostic Accuracy Studies Tool (QUADAS-2), including four main domains: patient selection, index test, reference standard, flow and timing [14].

## Results and discussion

**Fig. 1:**
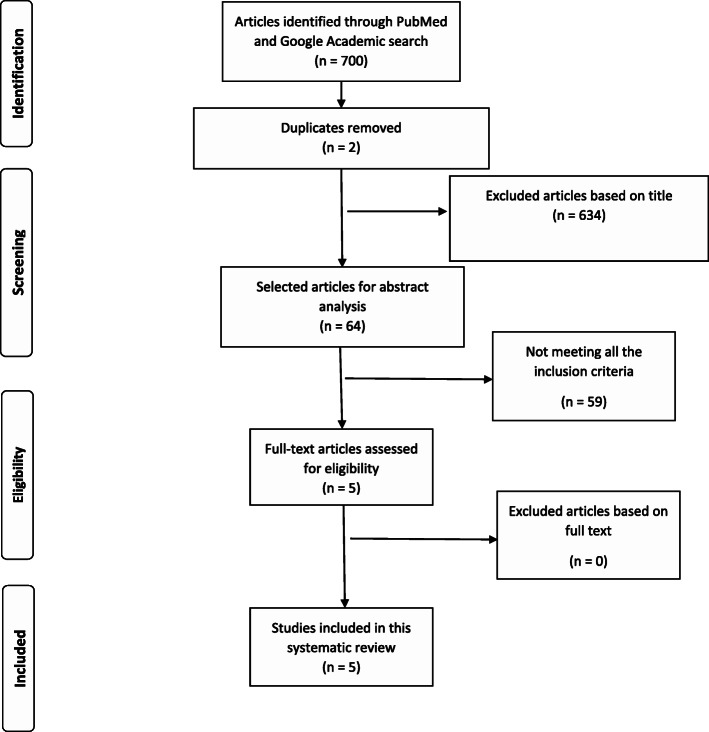
PRISMA 2009 flow diagram for head and neck cancer detection based on salivary exosomal microRNAs (from Moher D, Liberati A, Tetzlaff J, Altman DG, The PRISMA Group (2009) Preferred Reporting Items for Systematic Reviews and Meta-Analyses: The PRISMA Statement. PLoS Med 6(7): e1000097. 10.1371/journal.pmed.100009)

A total of 700 articles (687 on Pubmed.gov and 23 on Google Academic) resulted in the searching process. Two articles were duplicated and therefore excluded. A number of sixty-four original papers were selected for the screening stage of the abstract, the rest of the articles being excluded after title examination. Following the screening process, fifty-nine articles were removed from the analysis due to not meeting all of the aforementioned criteria. As a result, five articles were included for full-text analysis and review (Fig. 1), four focused on cancer and one on premalignant lesion detection (Table 1).

**Table 1:** Articles included for this systematic review and quantity of saliva required for exosomal miR detection

Author and publication year	Title	Study type	Saliva sample (ml)
Byun et al. 2015 [15]	Diagnostic profiling of salivary exosomal microRNAs in oral lichen planus patients	Saliva	5
Langevin et al. 2017 [16]	Comprehensive microRNA-sequencing of exosomes derived from head and neck carcinoma cells in vitro reveals common secretion profiles and potential utility as salivary biomarkers	Saliva and *in vitro*	2
Gai et al. 2018 [17]	Salivary extracellular vesicle-associated miRNAs as potential biomarkers in oral squamous cell carcinoma	Saliva	-
He et al. 2020 [18]	Salivary exosomal miR-24-3p serves as a potential detective biomarker for oral squamous cell carcinoma screening	Saliva, *in vitro* and tumor tissue	5
Farag et al. 2021 [19]	MiR-134/miR-200a-derived salivary exosomes are novel diagnostic biomarkers of oral squamous cell carcinoma	Saliva	3

“-” = unspecified

The study design was different between papers (Table 1). While three articles focused only on the examination of saliva samples, the other two included in vitro analysis of cell culture, among which, one specific research paper performed a supplementary examination on tissue sample. The studies including more analysis than saliva samples better explained the influence of miRs in head and neck cancer (HNC).

### Quality indicators

The articles were qualitatively assessed through QUEST review analysis. All papers obtained a strong qualificative after analysis, greater than 26, that classify them as high-quality studies. In addition, after QUADAS-2 analysis, all five articles presented a low bias risk and high applicability in the four parts of the test (Table 2).

**Table 2:** QUADAS-2 assessment of the risk bias and applicability for the included articles in this review

Study	Risk of bias				Applicability concerns		
	Patient selection	Index test	Reference standard	Flow and timing	Patient selection	Index test	Reference standard
Byun et al. [15]	**?**	**+**	**+**	**+**	**+**	**+**	**+**
Langevin et al. [16]	**+**	**+**	**+**	**+**	**+**	**+**	**+**
Gai et al. [17]	**+**	**?**	**+**	**+**	**+**	**+**	**+**
He et al. [18]	**+**	**+**	**+**	**+**	**+**	**+**	**+**
Farag et al. [19]	**+**	**+**	**+**	**+**	**+**	**+**	**+**

+ Low risk; - High Risk; **?** Unclear

### Saliva samples

Saliva is named by some authors “body mirror” due to the large amount of fingerprints of systemic diseases or tumors [20–22]. Besides exosomal miRs, other fingerprints are also present in the saliva. Nucleic acids (e.g. cell-free miR (cfmiR), mRNA, various types of DNA), proteins, cytokines (e.g. Il-8, Il-6, Il-10), circulant tumor cells (CTC) or other extracellular vesicles are examples of salivary biomarkers of tumors or systemic diseases [23, 24].

The presence of various biomarkers in saliva makes it relevant for liquid biopsy examination [25]. Moreover, saliva is a friendlier environment for various biomarkers. For example, the lower quantity of ribonuclease compared with blood reduces the degradation of miRs [26, 27]. Saliva is a cost-effective sample for cancer detection while the harvesting is easy and non-invasive, does not require trained personnel and transport, and deposit is less sensitive than for other types of samples [28, 29].

Exosomal miRs, like other biomarkers of cancer, are released by the tumor cells in the peritumoral environment, and they are further driven either in the blood stream or in the tumor lavage fluids, such as saliva [30, 31]. However, the salivary exosomal cargo originates from the lavage process and blood stream from where exosomes passively pass into saliva through gingival sulcus fluid. As a consequence, the concentration of exosomal miRs in saliva is higher than in blood [32].

The size of salivary samples used for exosomal miR identification varied in the papers reviewed, from 2 to 5 ml (Table 1) [15–19]. Still, Gallo et al. obtained exosomal miR from 1 ml of saliva, which proves that a small amount of saliva sample contains sufficient exosomal miR for a proper analysis [33].

### Isolation and characterization of Exosomes

**Fig. 2:**
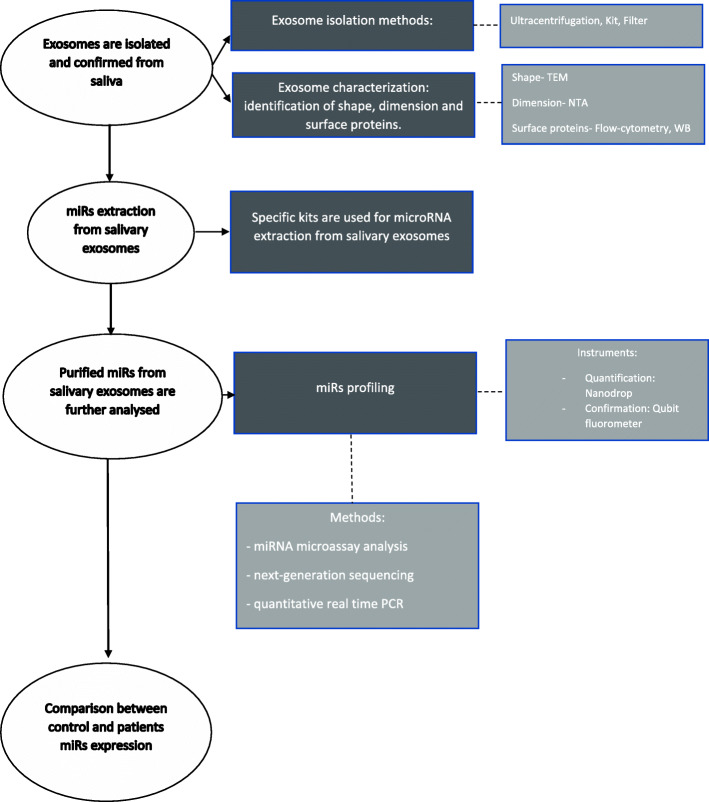
miRs’ extraction methods and quantification of their expression from salivary exosomes of the control and cancer patients

Exosomal miR analysis from saliva samples requires isolation and characterization of the exosomes and identification of miRs and quantification of their expression (Fig. 2). In the studies selected for this review, exosomes were isolated by ultracentrifugation (2 articles), by using extraction kits (2 articles) or by filters (1 article) (Table 3). Langevin et al. (2017), as well as Farag et al. (2021), used differential ultracentrifugation due to the bias that can appear in commercial affinity-based approaches (e.g. extraction kits) for isolation of cancer-associated exosomes [16, 19]. Differential ultracentrifugation is the golden standard for exosome isolation, and it consists of removal of viable cells, cellular debris and macromolecular proteins by centrifugation at different speeds, ended by 100,000 × g ultracentrifugation for 70 minutes to obtain the exosomes. This method is time-consuming, labor-intensive, and instrument-dependent, associated with a risk of exosome losing [25, 34]. He et al. (2020) and Byun et al. (2015) used a kit for exosome purification (ExoQuick-TCTM -SBI, Mountain View, CA, USA) and a protocol of centrifugation with reduced g force (1500), that is stated by the authors to be quicker, easier and more feasible for cancer screening [15, 18]. In contrast with the above-mentioned methods, Gai et al. (2018) used filters of 0.2 μm and 1500 × g protocol of centrifugation, which is also a short-time technique [17]. Even though all of the aforementioned protocols lead to exosome isolation and purification, different types of exosome isolation kits offer different amounts of exosomes and different dispersion stability. Therefore, these variations reduce reproducibility between measurements and cause inconsistency when it comes to result comparison from different laboratories [35, 36]. Exosome purification is a sensitive process and the results may be influenced by debris, bacterial flora and various types of sample harvesting (e.g. stimulated or unstimulated saliva) [25].

**Table 3:** Isolation and characterization of exosomes

Author	Exosome isolation	Exosome characterization
Langevin et al. (2017) [16]	Ultracentrifugation	TEM, NTA, WB (CD 81, TSG 101)
Farag et al. (2021) [19]	Ultracentrifugation	TEM
Byun et al. (2015) [15]	Kit	TEM, Flow cytometry
He et al. (2020) [18]	kit	TEM, NTA, negative staining, WB (CD81, CD63, TSG101)
Gai et al. (2018) [17]	filters	TEM, NTA, negative staining, WB (CD9, CD63, TSG101)

Characterization of the exosomes defines the physicochemical properties of exosomes, such as size, shape, surface charge, density and porosity [35]. In the papers reviewed, transmission electron microscopy (TEM), nanoparticle-tracking analysis (NTA), negative staining, flow cytometry and western blot (WB) were used for the confirmation stage of analysis. TEM was used in all of the articles included for this study. In addition to TEM, Byun et al. (2015) used flow cytometry by Exo-Flow kits (SBI) for the confirmation of the exosomes [15]. Langevin et al. (2017) used TEM for visual confirmation, WB for immunohistochemical confirmation and NTA for quantification of exosomes [16]. Additionally, He et al. (2020) and Gai et al. (2018) included negative staining to improve the characterization accuracy [17, 18]. NTA is a biophysical approach used for optical particle tracking performed for exosome characterization, that can measure the concentration and size distribution of vesicles in the 10 to 1000-nm range [35]. TEM is also a biophysical method that uses large objectives on microscopes, in a special and controlled environment, for describing the size, shape and aspect of the exosomes. For example, exosomes can be seen on TEM examination as 30–150-nm microvesicles, rounded or irregular, alone or clustered [35]. Farag et al. (2021) described exosomes as a heterogeneous population in oral squamous cell carcinoma (OSCC) and in tobacco smoking patients, presenting irregular morphology and various shapes and dimension (ranging from 30 to 400 nm) [19]. On the contrary, exosomes observed in healthy subjects’ saliva revealed homogeneous circular microvesicles with a size ranging from 60 to 90 nm. Additionally, large vesicle aggregates were observed in the OSCC samples compared with smokers’ samples [19]. These findings regarding exosome characterization were in accordance with Gai and Sharma’s results [17, 37]. Conversely, saliva of patients who suffer from premalignant lesions, such as oral lichen planus (OLP), proved to contain typical rounded vesicle structures sized 20 to 100 nm, more similar to healthy patients’ saliva [19]. Moreover, a large concentration of exosomes was discovered in cancer patients’ saliva compared with healthy subjects’ [19]. The explanation of the increased number of exosomes and the change in shape may be the tumor-associated hypoxia [26, 38].

Flow cytometry and WB are molecular approaches used to characterize the surface of the exosomes [35]. WB identified changes in the CD63, CD9, CD81, TSG101 and Alix surface proteins on the cancerous and premalignant exosomes. An increase in CD9 and CD81 and a decrease in CD63 proved to be markers of malignancy [25]. Recently, Gai et al. (2018) indicated that an increased presence of CD44 appears on the surface of salivary exosomes of cancer patients and is correlated with the changes of the miR-512-3p and 302b-3p expression [17].

### MicroRNAs in head and neck cancer

The next step after exosome isolation and characterization is miR extraction from exosomal pellet (Fig. 2). In the papers reviewed, different kits were used for miR purification from each isolated exosome (e.g. *Micro kit, Qiagen, Valencia, CA; TRIzol reagent, Invitrogen, Carlsbad, CA, USA; EXOs, Zymoresearch Quick-gRNA™ MiniPrep kit, USA; mirVana Isolation Kit, Thermo Fisher Scientific*). The purified miRs were further quantified and confirmed using specific protocols and instruments (e.g. NanoDrop for quantification and Qubit fluorometer for confirmation). The main miR profiling methods are next-generation sequencing, quantitative real-time PCR (qRT-PCR) and miRNA microassay analysis [26]. The expression of the potential miR was quantified by droplet digital PCR (ddPCR) and qRT-PCR. qRT-PCR was preferred (4 articles) for this stage of analysis (Table 4).

**Table 4:** MicroRNA types and mechanisms of action

Author	RNA analysis	microRNAs	Mechanisms of action
Byun et al. (2015) [15]	miRNA-Ma, qRT-PCR	miR-4484, miR-1246,1290	
Langevin et al. (2017) [16]	miRNA-seq, ddPCR	miR-10B-5p, miR-486-5p, miR-486-3p	TLR, FcR
Gai et al. (2018) [17]	qRT-PCR assay, qRT-PCR	miR-302b-3p, miR-517-3p, miR-512-3p, miR-412-3p	ErbB, TGF Beta signaling pathways, CD 44
He et al. (2020) [18]	miRNA-MA, qRT-PCR	miR-24-3p	PER 1
Farag et al. (2021) [19]	qRT-PCR	miR-134, miR-200a	PDCD7, EMT

miR dysregulation, as well as their presence or absence in tissues or biofluid samples, may represent an important diagnostic and prognostic factor for HNSCC. More specific, microRNAs not only reflect the changes induced by cancerous cells but may also represent the sophisticated instruments which sustain these changes. For example, in HNSCC, oncogenic miRs are upregulated, and they are responsible for targeting and silencing the tumor-suppressor genes which can modulate the outset, development and metastasis of cancerous cells. In contrast, tumor-suppressor miRs are downregulated, that reduce the modulation of oncogenes, sustaining the malignancy. Such an example is miR-200a reduction that facilitate epithelial-mesenchymal transition (EMT) by losing control over the modulation of *ZEB* genes [19].

In the reviewed papers, salivary exosomal miR-10b-5p, miR-486-5p, miR-486-5-3p [16], miR-24-3p [18], miR-134, miR-200a [19], miR-302b-3p, miR-517-3p, miR-512-3p and miR-412-3p [17] had different levels of expressions in head and neck cancer saliva samples compared with normal (Table 4). Significantly higher expression of some of the above-mentioned miRs were proved by the authors, among which, miR-24-3p is 5.73-fold elevated in cancer patients’ saliva compared with healthy samples [18]. Farag et al. (2021) showed a significant downregulation of miR-200a in cancer samples [19]. This result was in agreement with Park et al.’s finding in cfmiR 200a [23]. Besides the overexpression of miR-412-3p and miR-512-3p in salivary extracellular vesicles of oral cancer patients, Gai et al. (2018) proved also that miR-302b-3p and miR-517b-3p were solely present in oral cavity SCC saliva samples [17]. Likewise, miR-4484, miR-1246 and miR-1290 were differently expressed in OLP saliva samples [15]. miR-4484 was significantly upregulated in salivary exosomes of OLP patients with a range between 2- and 98-fold [15].

Regarding sensitivity and specificity for head and neck cancer detection, the authors obtained various results. The highest specificity (100%) was obtained by Langevin et al. (2017) by using miR-10b-5p, but it was associated with a very low sensitivity of 18% [16]. However, identification of both miR-10b-5p and miR-486-5p in saliva could discriminate cancer samples from healthy samples with an accuracy of 85% [16]. A more balanced result between sensitivity and specificity was obtained by He et al. (2020), who showed a sensitivity and specificity of 64.4% and 80% respectively, by using miR-24-3p [18]. Gai et al. (2018) also proved a high sensitivity and specificity expressed by maximum Younden’s index with AUC values of 0.847 and 0.871 for miR-512-3p and miR-412-3p respectively [17]. The statistical analysis was based on different cutpoints of miR expression. For example, miR-10b-5p had a cutpoint of > 1.0 copies/μL, and miR-486-5p had a cutpoint of > 100 copies/μL [16].

Salivary exosomal miR detection presents some advantages over other possible HNC biomarker-based detection. Salivary exosomal miRs can be detected in small amounts of saliva, and they can be identified either by the analysis of the whole saliva or selectively the supernatant [26, 31, 33]. Additionally, some of the HNC biomarkers proved to be site specific for the same pathological types. For example, miR 200 group and miR-485-5p proved to be specific for oral cavity SCC, and miR-10b-5p was associated with oropharynx SCC [16, 30]. More precisely, Lin et al. indicated the SCC of the tonsils as the specific site for miR-200 family [30].

Salivary exosomal miRs are correlated with the stage of the disease and the histopathological type and grade. While miR-134 expression was higher in high-grade OSCC, miR-200a expression was higher in low-grade tumors [19]. miR-486-5p was able to detect stage I of cancer, supporting that the salivary exosomal miRs are promising biomarkers for early-stage cancer detection [16].

In precancerous lesions, exosomal microRNAs can be used as tools for identification of the progression towards the malignant phase. miR-4484 and miR-10b-5p are examples that mark the transformation of OLP and oral dysplasia to OSCC [15, 26, 39]. Moreover, miR-200a was associated with smoking-induced epigenetic changes, and its lower expression was correlated with a higher risk of oral cancer development [19]. Besides pointing the malign transformation, miR-200a was significantly increased 12 months after radiotherapy treatment [40]. All these results indicate that salivary exosomal miRs are potential biomarkers for head and neck cancer monitorization and treatment evaluation.

HPV infection status can also be identified by miRs. MiR-486-5p was detected in salivary exosomes from p16-positive oropharyngeal SCC patients and controls, and miR-100-5p was associated with HPV-negative oropharyngeal SCC [16, 41]. Other miRs, such as miR-9 family, can discriminate between HPV-positive and -negative statuses associated with HNSCC [18, 41–43].

Some disadvantages of miR-based cancer detection can be related to non-cancerous conditions, such as smoking, inflammation and aging process, which may alter the miR expression and further interfere with the cancer diagnosis process [44]. For example, miR-24-3p is differently expressed in elderly compared to young adults [45]. The lack of a standardized protocol will lead to a lower concordance between results and further to a lack of validation between laboratories [33]. miRs proved to have various degradation times into different fluids. For example, miR-124a has rapidly decreased in saliva samples (less than 10% detectable level after 3 min) compared with miR-191 (approximately 30% detectable level after 30 min) [23, 46].

Different expression of a specific miRs in various histopathological types represent another disadvantage. For example, pancreatic ductal adenocarcinoma and oral and oropharyngeal SCC showed altered expression with different values of miR-200b. The role of miR-200b may represent an explanation. Functioning as regulators of gene expression, same miR may influence different biological pathways of two distinct malignant processes [21, 28]. Also, the presence of miR-20-5p in the exosomes from both head and neck and cervical HPV positive SCC, indicate that miR-20-5p is a marker of HPV-associated infection of SCC and the mechanism of carcinogenesis is similar [41, 43]. In addition, the fact that altered expression of one miR may offer various and totally different information is exemplified by miR-4484, which represents a biomarker for OLP and can also predict the lymph node metastasis of HSCC. The changes in the immune response induced by miR-4484 may justify the superposition of this miR activity in the two pathologies [17].

### MicroRNA pathways of carcinogenesis

Salivary exosomal miRs detain various mechanisms of action which can sustain tumor microenvironment and thus, contribute to the proliferation of tumor cells (Table 4). miR-486-5p and miR-10b-5p are involved in the host’s immune response through negative modulation of targets, such as tool-like receptors (TLR) and Fc receptors [47, 48]. In OSCC, TLR favor tumor progression and chemotherapy resistance especially through the recruitment of suppressive regulatory T cells [49]. Another miR that induces drug resistance (Tamoxifen) is miR-24-3p, which can also cause a shortening of the cell cycle and an increase in the proliferation rate of tumor cells and increase the efficacy of cell colony forming through *PER1* pathway [50]. By directly targeting 3′-UTR of *PER1*, which is involved in cancer antiproliferative effect, miR-24-3p inhibits its expression and lock the cell cycle [51]. Thus, these mechanisms explain the correlation between increased expression of miR-24-3p and decreased *PER1* expression in HNSCC [18]. However, the mechanism of action of 24-3p is still debated in scientific literature, on different types of cancers, such as lung or breast [52, 53].

In contrast with the above-mentioned miRs, elevated levels of miR-134 increase growth and migration of tumor cells by reducing the *E-cadherin* expression and, further, interacting with *Programmed Cell Death 7 (P7DC7)* in OSCC [19, 54]. In addition, miR-134 inactivates the *WWOX* tumor suppressor genes contributing repeatedly to carcinogenesis. These pathways explain the poor survival rate of patients with OSCC, which was associated with high expression of miR-134 [54]. On the other hand, Salazar et al.’s results indicated a lower expression of miR-134 in OSCC saliva samples [55]. This inadvertence may be caused not only by the different sample examination, cell culture and saliva, but also by the contrasting method of quantification. While in Shih-Yuan Peng et al.’s study, miR-134 expression was upregulated after a qRT-PCR analysis, in Salazar et al., research miR-134 was found downregulated in the microarray data set [45, 55].

Salivary miR-10b and miR-200a are remarkable examples of tumor invasion and metastasis biomarkers [19, 32, 56]. An increase in miR-10b expression is a marker of progression from premalignant lesion to OSCC. Due to miR-10b involvement in oncosuppression, elevated levels of miR-10b may represent protective actions taken by the normal cells against malign transformation [57]. Furthermore, this biomarker may allow to distinguish between progressing or non-progressing oral low-grade dysplasia to OSCC [39]. miR-200a favors tumor invasion, metastasis and resistance to cancer therapy. Being classified as a tumor-suppressor miR that preserves epithelial phenotype, miR-200a inhibits EMT, tumor invasion and metastasis generation [36, 58, 59]. Consequently, the downregulation of miR-200a permits EMT and negatively influences host response against tumor expansion, and thus, it can be used as a marker of malignant transformation and invasion [23, 60]. Additionally, miR-200a is involved in the DNA methylation induced by tobacco smoking, that leads to progressive accumulation of multiple genetic abnormalities and further to development of OSCC [60, 61]. Moreover, miR-200 family is involved in *ZEB1* and *ZEB2* modified expression and in lytic replication of Epstein-Barr virus (EBV) in B cells in the EBV pathophysiology [30, 58].

Compared to above-mentioned miRs, miR-512-3p and miR-27a-3p are involved in *ErbB* signaling, which promotes cell proliferation and tumor survival [17, 62]. Additionally, they increase the expression of CD 44, which promotes *ERK1/2* phosphorylation and cancer invasiveness [63]. miR-512-3p, miR-412-3p, miR-27a-3p, and miR-302b-3p are also involved in targeting genes of the *TGFβ* signaling pathway and Bmi1, that promote cells formation, migration and metastasis [64, 65]. miR-27a-3p overexpression reduces the control of EMT through *YAP* and *MCPH 1* modification [66].

Exosomal miRs from saliva samples seem to differentiate patients with oral and oropharyngeal cancer and premalignant lesion from healthy subjects [15–19] even in early stages [67]. The technologies used for the detection of miRNAs required less time and lower costs compared with other biomarkers, such as proteins. Also, the fact that miRNAs have a high degree of specificity and can be associated with cancer morpho-functional changes, gives these RNA molecules the advantage of being used as markers not only for the detection, but also for monitoring the progression of the disease [68]. MicroRNAs present intricate and various mechanisms of action, which indicate the wide involvement in malignancy patterns and also make them a confident fingerprint of cancer.

The majority of cancer associated miRs are concentrated in saliva and serum exosomes [33, 69]. Exosomes represent a supplementary barrier against miR enzymatic degradation due to their lipidic double layer. Moreover, exosomal miR originates from an active process of carcinogenesis, which aims to control the environment for tumor expansion [70]. Contrarily, cfmiRs can be passively released by dead and desquamated cells and can consequently induce an increased rate of false negative results in cancer detection [23, 31, 71].

Exosomes are independent biomarkers of cancer due to the aforementioned cancer-related changes in concentration, size, shape and aspect, as well as various expression of surface proteins. Based on these modifications, exosomes can individually diagnose the histopathological grade of tumor and can represent a prognostic marker for metastasis due to exosomes roles in intercellular communication, tumor proliferation and cellular growth. Exosomal changes reverse after treatment [19, 26, 27, 38]. Nevertheless, alcohol consumption induces changes in exosome characteristics, fact to be mentioned and taken into consideration for exosome evaluation [18, 60].

Given the above, the additional step of exosome isolation increases the accuracy of exosomal miRs based HNC diagnosis. Ultracentrifugation proves to be more predictable and induces less interobserver differences compared with the other methods for exosomal isolation. TEM, NTA and WB are the most reliable methods for salivary exosome characterization and flow cytometry and negative staining can point out additional information.

Detection of exosomal miRs from saliva samples is non-invasive, comfortable for the patient and less sensitive for harvesting and transport compared with other biomarkers, such as mRNA. It also has a robust stability and resistance to degradation in body fluids [26]. Moreover, exosomes are found in abundance in saliva sample compared to other biological fluids and they can be isolated from small amounts of liquid [27, 46]. Given the above, saliva is a promising liquid biopsy for mass screening and rapid detection of head and neck cancer.

We think that this systematic review met the scope of identifying the most reliable exosomal microRNAs in saliva samples and bring forward the methods of analyzing these biomarkers for head and neck cancer detection, in order to help future research in choosing the proper saliva processing method and the specific exosomal miRs for head and neck squamous cell carcinoma identification.

## Conclusions

Exosomal miR-10b-5p, miR-486-5p, miR-24-3p and miR-200a are the most promising biomarkers for oral and oropharyngeal cancer detection identified in saliva samples. Moreover, miR-486-5p can be detected from early stages. Salivary miRs purification from exosomes improves the diagnosis accuracy due to the increased concentration of miRs in exosomes and the supplementary protection that exosomes offer to the nucleic acids. Saliva is a proper sample for rapid and non-invasive cancer diagnosis and patient’s follow-up.

## Data Availability

Not applicable.

## Abbreviations

miRNA: MicroRNA
HNSCC: Head and neck squamous cell carcinoma
EMT: Epithelial–mesenchymal transition
EV: Extracellular vesicles
MVB: Multivesicular bodies
RISC: RNA-induced silencing complex
3’UTR: 3’ Untranslated region
QUEST: Quality Evaluation Scoring Tool
CTC: Circulant tumor cells
cfmiR: Cell-free miR
TEM: Transmission electron microscopy
NTA: Nanoparticle-tracking analysis
WB: Western blot
OSCC: Oral squamous cell carcinoma
OLP: Oral lichen planus
qRT-PCR: Real-time polymerase chain reaction
TLR: Tool-like receptors
